# Rapid Construction of a Replication-Competent Infectious Clone of Human Adenovirus Type 14 by Gibson Assembly

**DOI:** 10.3390/v10100568

**Published:** 2018-10-18

**Authors:** Haibin Pan, Yuqian Yan, Jing Zhang, Shan Zhao, Liqiang Feng, Junxian Ou, Na Cao, Min Li, Wei Zhao, Chengsong Wan, Ashrafali M. Ismail, Jaya Rajaiya, James Chodosh, Qiwei Zhang

**Affiliations:** 1 Guangdong Provincial Key Laboratory of Tropical Disease Research, School of Public Health, Southern Medical University, Guangzhou 510515, China; perbin0112@gmail.com (H.P.); yyq@smu.edu.cn (Y.Y.); 243562394@smu.edu.cn (J.Z.); zhaoshan1104@smu.edu.cn (S.Z.); ojx426@smu.edu.cn (J.O.); caona1125@smu.edu.cn (N.C.); limin930701@gmail.com (M.L.); zhaowei@smu.edu.cn (W.Z.); gzwcs@smu.edu.cn (C.W.); 2 Guangzhou Institutes of Biomedicine and Health, Chinese Academy of Sciences, Guangzhou 510530, China; feng_liqiang@gibh.ac.cn; 3 Department of Ophthalmology, Howe Laboratory, Massachusetts Eye and Ear, Harvard Medical School, Boston, MA 02114, USA; mohamed_ismail@meei.harvard.edu (A.M.I.); jaya_rajaiya@meei.harvard.edu (J.R.); james_chodosh@meei.harvard.edu (J.C.)

**Keywords:** Human adenovirus type 14, infectious clone, Gibson Assembly

## Abstract

In 1955, Human adenovirus type 14 (HAdV-B14p) was firstly identified in a military trainee diagnosed as acute respiratory disease (ARD) in the Netherlands. Fifty years later, a genomic variant, HAdV-B14p1, re-emerged in the U.S. and caused large and fatal ARD outbreaks. Subsequently, more and more ARD outbreaks occurred in Canada, the UK, Ireland, and China, in both military and civil settings. To generate a tool for the efficient characterization of this new genomic variant, a full-length infectious genomic clone of HAdV-B14 was successfully constructed using one-step Gibson Assembly method in this study. Firstly, the full genome of HAdV-B14p1 strain GZ01, the first HAdV-B14 isolate in China, was assembled into pBR322 plasmid by Gibson Assembly. The pBRAdV14 plasmid, generated by Gibson Assembly, was analyzed and verified by PCR, restriction enzymes digestion and the sequencing. Secondly, viruses were rescued from pBRAdV14-transfected A549 cells. The integrity of the rescued viruses was identified by restriction enzyme analysis. The complete sequence of the infectious clone was further sequenced. No mutation was found in the infectious clone during the construction when compared with the parental virus and pBR322 sequences. The direct immunofluorescence assay indicated the expression of the hexon protein. Finally, typical virions were observed; the one-step growth curves further showed that the DNA replication and viral reproduction efficiency of pBRAd14 derived viruses was similar with that of wild-type HAdV-B14 strain. The successful construction of the replication-competent infectious clone of pBRAdV14 facilitates the development of vaccine and antiviral drugs against HAdV-B14, as well as provides a novel strategy for rapid construction of infectious viral clones for other large-genome DNA viruses.

## 1. Introduction

Human adenoviruses (HAdVs) are non-enveloped and double-strand DNA viruses which have a diameter of 70–90 nanometers and a genome of 35–40 kb. Currently, 90 genotypes have been identified and classified into seven species (A to G), in which types 1–51 were initially characterized according to serum neutralization and hemagglutination-inhibition tests, whereas types 52 and the other following types were identified using the whole-genome sequence as the typing standard [1,2,3,4,5,6,7]. All original serotypes have now been whole-genome sequenced. Species B HAdVs, as well as HAdV-E4 cause epidemics of a similar syndrome, acute respiratory disease (ARD) [8]. HAdV-B has been further divided into two subspecies (B1 and B2) according to their genome identities and restriction enzyme digestion patterns [9], and also the tissue tropism difference. HAdV-B3, -B7, -B16 and -B21 in HAdV-B1 are respiratory pathogens and can infect the respiratory tract; HAdV-B50, another member in HAdV-B1, does not cause a specific disease. HAdV-B11, B34 and B35 are in subspecies B2 and are recognized as renal or urinary tract pathogens. However, the other two members in HAdV-B2, HAdV-B14 and B55, are identified as respiratory pathogens [8,10,11,12].

In 1955, HAdV-B14 was firstly identified in a military trainee diagnosed as ARD in the Netherlands [13]. In the same year, the second case was reported in a civilian setting in England (1955) [14]. Then, after approximately 50 years of silence, a sudden epidemic of HAdV-B14 infections occurred at the military recruit training centers throughout the United States [15]. Soon the outbreaks of HAdV-B14–associated febrile respiratory illness in US were more and more common among adults in both civilian and military settings [16,17,18,19,20,21]. In an outbreak associated with significant morbidity in Oregon, 60% of the patients were infected by HAdV-B14 and of these, 76% required hospitalization and 18% died [22]. This was a new genome type, HAdVB14p1. It further emerged and caused two ARD outbreaks in Ireland (2009) and the UK (2011), and led to the highest fatality rates reported for HAdV-B14 (33% and 23%, respectively) [16,17,18]. In 2011, HAdVB14p1 re-emerged in Canada and caused three patients to be hospitalized and one death [23]. In China in 2010, two HAdV-B14p1 isolates emerged in Guangzhou and Beijing in October and December, respectively [8,11,24]. Subsequently, HAdV-B14p1 caused at least three additional ARD outbreaks in China: One occurred in an elementary school of Gansu Province (2011) with 43 students presenting with febrile respiratory illnesses [20], one occurred in Beijing (2012) with 30 adults hospitalized [24], and the third occurred in a middle school in Liaoning Province (2012) with 24 students presenting with febrile respiratory illnesses [25]. Given the severity and the rapid transmission of HAdV-B14 infections world-wide, it is critical to determine the biological characteristics and pathogenic mechanism of this novel genome type.

To generate a tool for the efficient characterization of HAdV-B14, a full-length infectious genomic clone of HAdV-B14 was successfully constructed using Gibson Assembly method in this study. Gibson Assembly is an isothermal, single-reaction method. It can assemble multiple overlapping DNA molecules easily by the concerted action of Phusion DNA polymerase, T5 exonuclease and Taq DNA ligase [26]. The cycle product is enriched in an isothermal system. In the reaction, T5 exonuclease removed nucleotides from 5′ termini, producing single-stranded 3′ overhangs in the adjacent fragments previously prepared by PCR with overlapping ends; Phusion DNA polymerase supplemented the gaps after annealing two fragments with overlaps, and Taq DNA ligase sealed the nicks [26,27,28]. This robust one-step DNA assembly method is simple, highly efficient, and is suitable for rapidly inserting long DNA fragments into vectors independent of restriction enzyme sites. It has been applied to seamlessly construct genes, genetic pathways and even the full-length genomes. In this study, we generated an infectious genomic clone of HAdV-B14 by Gibson Assembly, which will facilitate the development of vaccine and antiviral drugs against HAdV-B14, as well as provide a novel strategy for the rapid construction of infectious viral clones for other DNA viruses. 

## 2. Materials and Methods

### 2.1. Cells, Bacteria, Viruses, Plasmid and Enzymes

A549 cells were preserved in our lab (purchased from ATCC: CCL-185™). DH5α competent cells and plasmid pBR322 were purchased from TAKARA Co. (Dalian, China). The first HAdV-B14 strain (GZ01) in China, was isolated in our laboratory from a 17-month-old infant with tonsillitis (October 2010) [8,11]. Q5 DNA polymerase, T5 exonuclease, Phusion DNA polymerase, Taq DNA ligase and restriction enzymes were purchased from New England Biolabs (Ipswich, MA, USA). The cells were grown in Dulbecco’s modified Eagle’s medium (DMEM) plus 10% fetal bovine serum (Gibco) at 37 °C, supplemented with 5% CO_2_. Fluorescent quantitative PCR kit for HAdVs was purchased from Guangzhou Huayin Corp (Guangzhou, China). FITC-conjugated HAdV-14 monoclonal antibody (5F11) directly against HAdV-14 hexon was purchased from Guangzhou HuYanSuo Medical Technology (Guangzhou, China), which was produced in vivo in BALB/c mice using hybridoma technology and purified by Thermo Scientific Pierce Protein A/G Magnetic Beads.

According to the original Gibson Assembly protocol [26], 5× isothermal reaction buffer and the assembly master mixture were prepared. 5× isothermal reaction buffer contains 3 mL of 1 M Tris-HCl pH 7.5, 150 µL of 2 M MgCl_2_, 600 µL of 10 mM dNTP, 300 µL of 1 M DTT, 1.5 g PEG-8000 and 300 mL of 100 mM NAD. The assembly master mixture contains 40 µL 5× isothermal reaction buffer, 0.2 µL of 10 U µL^−1^ T5 exonuclease, 2.5 µL of 2 U µL^−1^ Phusion DNA polymerase, 20 µL of 40 U µL^−1^ Taq DNA ligase, and water, with a final volume of 150 µL. All reagents were stored at −20 °C.

### 2.2. Construction and Screening of Recombinant Infectious Clones of HAdV-B14 by Gibson Assembly Method

**Figure 1:**
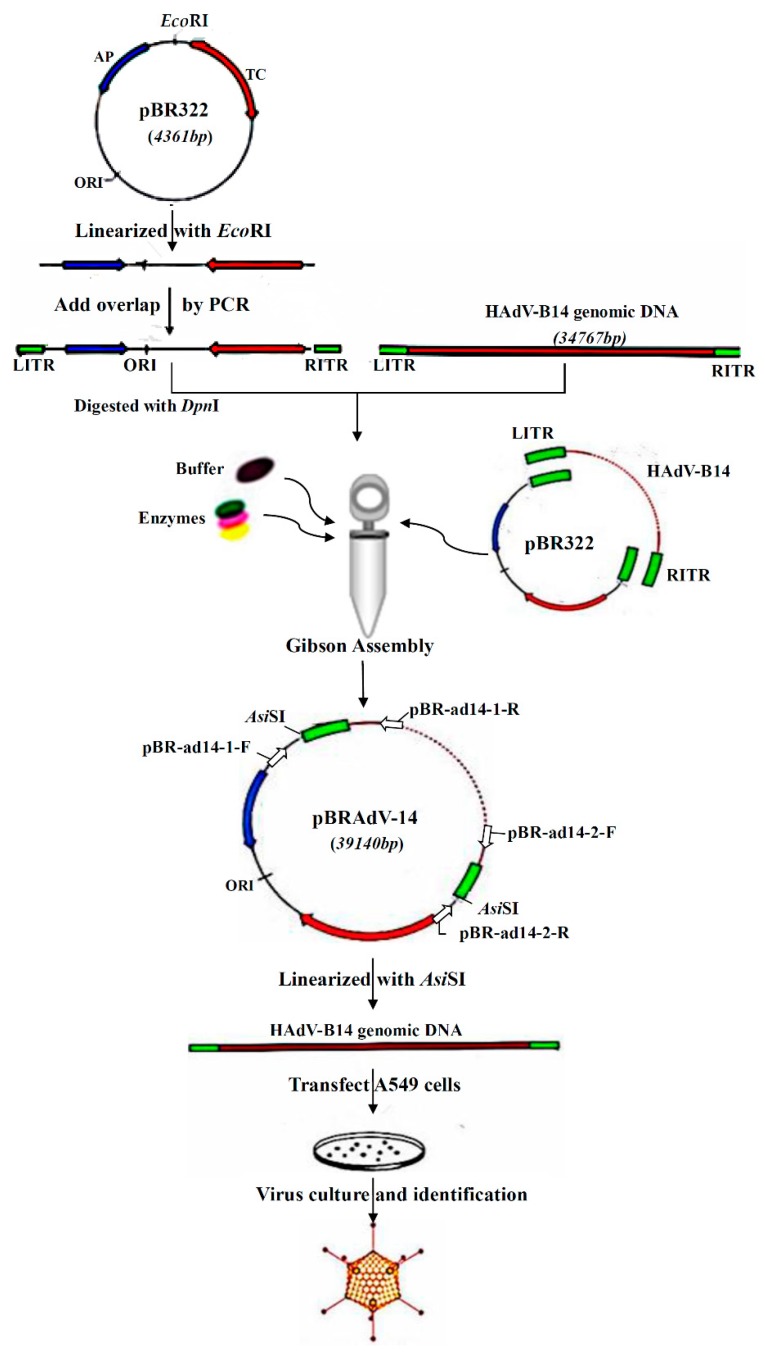
The schematic diagram of the construction of an infectious clone of HAdV-B14.

Due to the advantages of operational simplicity, high efficiency, seamless splicing, and large assembly scale (the reported maximum molecular scale of splicing is 583 kb) [26], Gibson assembly provides a new strategy for the construction of human adenovirus infectious clones. Wild-type HAdV-B14p1 isolate GZ01 was amplified in A549 cells. Adenoviral genomic DNA was extracted from the viral culture using the modified conventional adenovirus genome extraction method [29]. The linearized pBR322 plasmid digested by *Eco*RI served as PCR template and was PCR amplified by Q5 DNA polymerase, using primers that contained 40 bp Inverted Terminal Repeat (ITR) sequence of HAdV-B14 and *Asi*SI sites at both 5′ ends of the primers (Table 1). The plasmid DNA template in the PCR products was eliminated with *Dpn*I enzyme. Then, the HAdV-B14 genomic DNA and purified PCR product were mixed in a tube in a ratio of 4:1. After that, the assembly master mixture of 15 µL was added into the tube, and incubated at 50 °C for 1 h. The resultant Gibson reaction products were transformed into DH5α competent cells and cultured in the *Amp*^+^ LB plates overnight. Positive clones were screened by PCR using two pairs of primers, of which, primers pBR-ad14-1-F and pBR-ad14-1-R targeted the right end of PBR322 and the left terminal of viral genome, respectively, while primers pBR-ad14-2-F and pBR-ad14-2-R targeted the right terminal of viral genome and left end of pBR322 (Table 1 and Figure 1). The positive plasmid was extracted and the size was confirmed by electrophoresis. The plasmid was further screened by PCR using primers targeting HAdV-B14 hexon (hypervariable region) (HVR-F, HVR-R), penton base (Penton-F, Penton-R), and fiber genes (Fiber-F, Fiber-R) (Table 1). Finally, the plasmid containing the infectious molecular clone of HAdV-B14 was successfully constructed by Gibson Assembly.

### 2.3. Rescue and Amplification of HAdV-B14 Infectious Viral Particles

To determine the infectivity of pBRAdV14, the positive plasmid DNA was digested with *Asi*SI, which targets the restriction sites at the both ends of HAdV-B14 genome. After digestion, the linearized HAdV-B14 genomic DNA released from the pBRAdV14 plasmid was transfected into A549 cells using Lipofectamine 2000 (Invitrogen, Carlsbad, CA, USA) according to the manufacturer’s instructions. At Day 5 post-transfection, A549 cells were subjected to three rounds of freezing and thawing. The harvested culture was used to infect A549 cells again, and the cells were cultured in DMEM plus 2% FBS. When cytopathic effect (CPE) was observed, the culture was harvested and stored in −80 °C freezer.

### 2.4. Restriction Enzyme and Complete Plasmid Sequencing Analysis of the Infectious Clone pBRAdV14 and the Adenoviral Genome

To identify whether the plasmid clones pBRAdV14 screened were correct or if any deletions/insertions occurred during the recombination and viral rescue, restriction enzyme analysis of the plasmid pBRAdV14 and the pBRAdV14–derived viral genomic DNA was performed. The pBRAdV14 plasmids, which were extracted by Axygen Plasmid Kit (Axygen, Union City, CA, USA), were digested with five restriction endonucleases, *Bam*HI, *Eco*RI, *Hind*III, *Sal*I and *Xba*I, respectively. Then the digested product was analyzed by agarose gel electrophoresis. A549 cells were transfected with linearized HAdV-B14 genomic DNA released from *Asi*SI digestion. When CPE was obvious, viral genomic DNA was extracted by the modified adenovirus genomic DNA extraction method [30]. The viral DNA was also digested and analyzed with the five restriction endonucleases used for the plasmid pBRAdV14. The complete sequence of the infectious clone pBRAdV14 was sequenced by Sanger primer-walking method with an average of three- to five-fold coverage of the plasmid sequence. The assembled sequence was aligned with the wild-type HAdV-B14 genomic sequence (GenBank accession no. JQ824845) and the pBR322 sequence to confirm if there was any mutation or not during the construction.

### 2.5. Direct Immunofluorescence and Fluorescence Forming Assay

A549 cells grown in a 12-well or 96-well plate were infected with pBRAdV14 derived viruses and wide-type HAdV-B14 strain GZ01, respectively. The viral culture was 10-fold diluted in series before infection, and the blank control group was also set. After 40–48 h culture at 37 °C and 5% CO_2_, the culture medium was removed and 0.5 mL cold methanol was added into each well and the cells were fixed at −20 °C for 10 min. The cells were rinsed three times with PBS containing 1% bovine serum albumin (BSA-PBS) for 5 min each time. At the fourth time, the cells were incubated with BSA-PBS for 30 min. After discarding BSA-PBS, the cells were incubated with FITC-conjugated HAdV-B14 monoclonal antibody (1:100 dilution) and shaken gently at 37 °C for 1 h, and then washed three times with BSA-PBS. After the final washing, the cells were examined and photographed under an inverted fluorescence microscope (Nikon Eclipse TE2000-U, Nikon, Tokyo, Japan) at the excitation and emission wavelength of 470 nm and 515 nm, respectively. The number of green positive cells in the appropriate dilution, which were in the range of 50-100 per well, was counted and quantified by fluorescence forming units (FFU) per ml of the viruses. The assay was performed in triplicate for reproducibility.

### 2.6. One-Step Growth Curves of HAdV-B14

To assess the viral replication efficiency of pBRAdV14–derived viruses, quantitative PCR (Q-PCR) was performed using SYBGreen real-time PCR kit with hexon-based plasmids as standards (Huayin Corp.; Guangzhou, China). A549 cells were cultured in 12-well plates and inoculated with 1×10^7^ DNA copies (at an MOI of 0.02) of parental HAdV-B14 strain GZ01 and pBRAdV14–derived viruses, respectively. The cells were harvested at 12, 24, 36, 48, 60, 72, 84, 96 h post infection and the viral genomic DNA copy numbers were determined via Q-PCR. Data analyses were performed with *MxPro* *Q*-*PCR* Software (Agilent, Santa Clara, CA, USA). The infectious viral particles (FFU/mL) in harvested viral culture at each time point were also quantified by fluorescence forming assay using FITC-conjugated HAdV-B14 monoclonal antibody, as described above. One-step growth curves were drawn using GraphPad Prism 5 (GraphPad Software Inc., San Diego, CA, USA).

### 2.7. Electron Microscopic Observation

The A549 cells infected with pBRAdV14–derived viruses were harvested at 72 h post-infection. After centrifugation at 10,000 g for 10 min, the virus suspension was dropped onto a piece of parafilm. A grid covered with carbon support formvar film was floated on the virus suspension for 1 min, then dropped into sodium phosphotungstate (pH 7.0) and exposed to UV radiation for 20 min. Finally, the adenovirus particles were observed under a FEI Tecnai-12 electron microscope (Hillsboro, OR, USA).

## 3. Results

### 3.1. Identification of Infectious Clones of HAdV-B14 and Rescue of the Viral Particles

**Figure 2:**
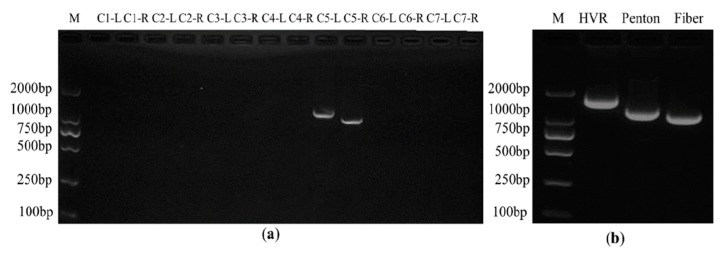
Identification of infectious clones of PBRAdV14 by PCR. (**a**) PCR screening with primers targeting the left ligation region. L: PCR with pBR-ad14-1-For and pBR-ad14-1-Rev; R: PCR with pBR-ad14-2-For and pBR-ad14-2-Rev. Seven clones were screened first, C1, C2, C3, C4, C5, C6, C7; (**b**) PCR screening with primers targeting the three major capsid genes: HVR, Fiber and Penton base. Length of positive PCR product is 1685 bp for HVR, 1253 bp for Penton base and 1153 bp for Fiber.

**Figure 3:**
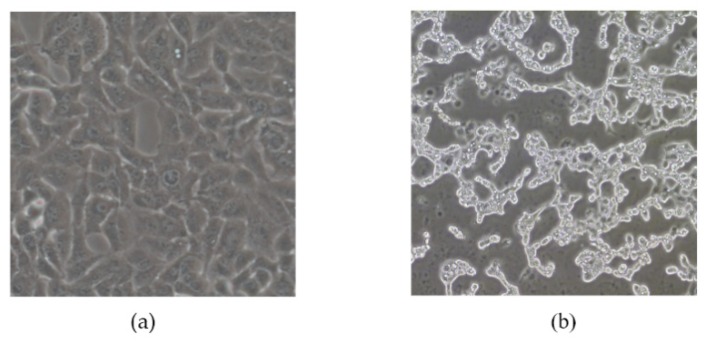
(**a**) The MOCK A549 cells; (**b**) A549 cells infected with pBRAdV14–derived viruses. CPE was observed (200×).

The infectious clones of plasmid pBRAdV14 were generated by the ligation of the PCR product of linearized pBR322 and HAdV-B14 genomic DNA at 50 °C for 1 h (Figure 1). Twelve bacterial clones were obtained and screened by PCR using two pairs of primers which target both ends of the adenoviral genome. Three clones were identified as PCR positive (Figure 2a; partial image of PCR products is shown). The three positive clones were further screened using primers targeting HAdV-B14 hexon, penton base and fiber genes (Figure 2b). Plasmid pBRAdV14 was linearized with *Asi*SI and the HAdV-B14 genome was released, which was then used to transfect the A549 cells. At 96 h post transfection, the cells were harvested and ruptured by freezing and thawing three times. The harvested virus culture was inoculated into A549 cells and cultured and harvested after 96 h. After two rounds of blind passage, obvious CPE was observed (Figure 3) and the virus was harvested and stored at −80 °C.

### 3.2. The Identification of the Genomic Sequences of pBRAdV14 and Plasmid Derived Viruses

**Figure 4:**
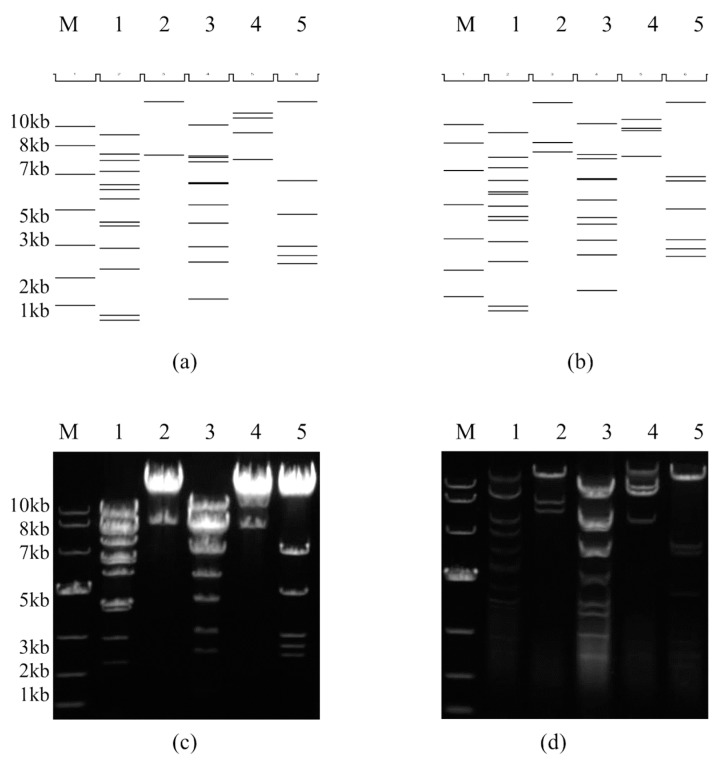
Restriction enzyme analysis of plasmid pBRAdV14 and the genome of pBRAdV14–derived viruses *in silico* and wet-bench. M: 10kb DNA ladder; Lane 1: *Bam*HI, Lane 2: *Eco*RI, Lane 3: *Hind*III, Lane 4: *Sal*I, Lane 5: *Xba*I. *In silico* (**a**) and wet-bench (**c**) restriction maps of pBRAdV14 were shown. *In silico* (**b**) and wet-bench (**d**) restriction maps of the genome of pBRAdV14 derived viruses were also shown. The predicted molecular weights of digested fragments of plasmid pBRAdV14 are 8559, 5927, 5233, 4225, 3251, 2987, 2470, 1575, 1573, 1456, 941, 612, 184, 156 bp for *Bam*HI, 333,25, 5815 bp for *Eco*RI, 10,314, 5692, 5569, 5106, 3391, 3334, 2208, 1545, 973, 709, 299 bp for *Hind*III, 13,025, 11,863, 8926, 5326 bp for *Sal*I, 31,279, 3542, 1843, 981, 811, 684 bp for *Xba*I. The predicted molecular weights of digested fragments of the viral genome are 8559, 5233, 4225, 3251, 2579, 2470, 1935, 1575, 1573, 1456, 941, 184, 156 bp for *Bam*HI, 21,923, 7029, 5815 bp for *Eco*RI, 10,279, 5569, 5106, 3391, 3334, 2208, 1545, 1354, 973, 709, 299 bp for *Hind*III, 11,206, 9309, 8926, 5326 bp for *Sal*I, 23,674, 3542, 3232, 1843, 981, 811, 684 bp for *Xba*I.

To identify whether there was any mutation in genomic sequences between the pBRAdV14 derived viruses and wild-type HAdV-B14, the restriction enzyme analysis *in silico* and wet-bench was performed (Figure 4). When compared with the *in silico* restriction maps produced by the Vector NTI 11.5.1 software (Invitrogen Corp.; San Diego, CA, USA), the restriction profiles of the pBRAdV14 and pBRAdV14–derived viruses were consistent with that produced by the software (Figure 4a,c and Figure 4b,d), and also consistent with the restriction profiles of parental wildtype GZ01 strain [8]. To further accurately identify in higher resolution if there were any mutations, deletions or insertions in the infectious clone during the construction, the complete sequence of pBRAdV14 was sequenced by Sanger primer-walking method. The alignment between the sequencing data and HAdV-14 genome and pBR322 sequences showed that there was no mutation in pBRAdV14. *Asi*SI sites were successfully added at both ITR ends of pBRAdV14 (gcgatcgcCATCATCAATAA). These results demonstrated that the infectious clone pBRAdV14 was stable and can be truly representative of the parental virus.

### 3.3. Direct Immunofluorescence Assay

**Figure 5:**
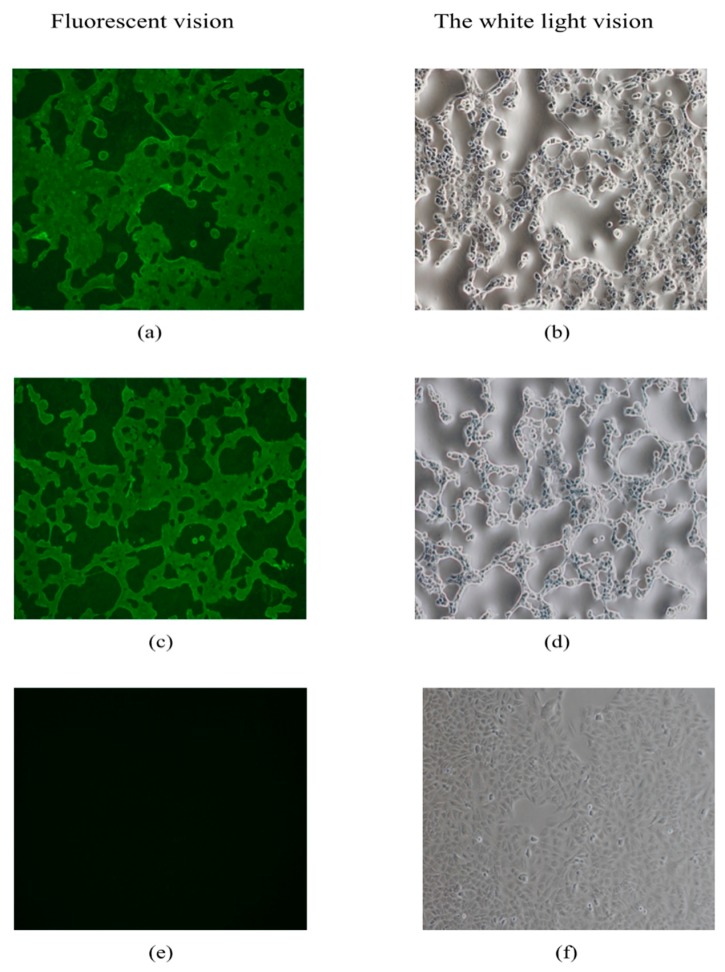
Direct immunofluorescence assay for the detection of the hexon protein of pBRAdV14 derived viruses. (**a**,**b**): The A549 cells infected with parental HAdV-B14 strain GZ01. (**c**,**d**): The A549 cells infected with the pBRAdV14 derived viruses. (**e**,**f**): MOCK control group without virus infection. (**a**,**c**,**e**) are in fluorescence vision; (**b**,**d**,**f**) are in the white light vision (100×).

To identify if there is viral replication and hexon gene expression of pBRAdV14–derived viruses, a direct immunofluorescence assay was performed. A549 cells were transiently infected with pBRAdV14 derived viruses and wide-type GZ01 strain, respectively. CPE appeared in A549 cells at 40 h after infection with the pBRAdV14 derived viruses and GZ01 (Figure 5b,d), and green fluorescence was also observed by immunostaining (Figure 5a,c). No green fluorescence was detected in the MOCK group (Figure 5e). 

### 3.4. Growth Characteristics of pBRAdV14 Derived Virus

**Figure 6:**
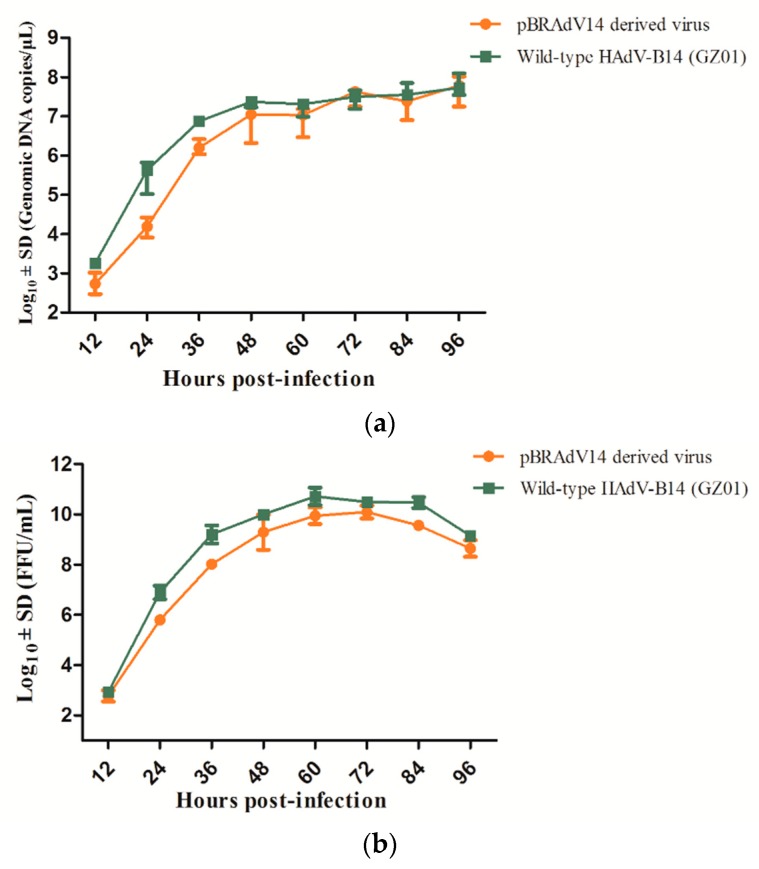
The one-step growth curves of pBRAdV14–derived viruses and parental HAdV-B14 GZ01 strain. Cells were harvested at 12, 24, 36, 48, 60, 72, 84 and 96 h post-infection of these two different origins of viruses at an MOI (multiplicity of infection) of 0.02. (**a**) Viral genomic DNA copy numbers in harvest viral culture were determined by quantitative PCR with Q-PCR kit (Huayin Corp.; Guangzhou, China); (**b**) infectious viral particles were quantified by fluorescence forming units (FFU) per ml as described in Materials and Method.

The replication efficiency of pBRAdV14–derived viruses and parental GZ01 strain were compared by quantification of genomic DNA using real-time PCR method (Figure 6a), as well as the infectious viral particles on the basis of FFU (Figure 6b). The result was shown by two one-step growth curves in Figure 6. As a whole, the DNA replication efficiency of pBRAdV14–derived viruses was highly similar to that of the parental HAdV-B14 strain GZ01, whereas the infectious viral reproduction from pBRAdV14–derived viruses was slightly lower than wild-type HAdV-14 isolate. Both viruses reached the peak of DNA replication at about 48 h after infection, whereas they reached the peak of infectious viral reproduction at 60 h after infection.

### 3.5. Electron Microscopic Observation

**Figure 7:**
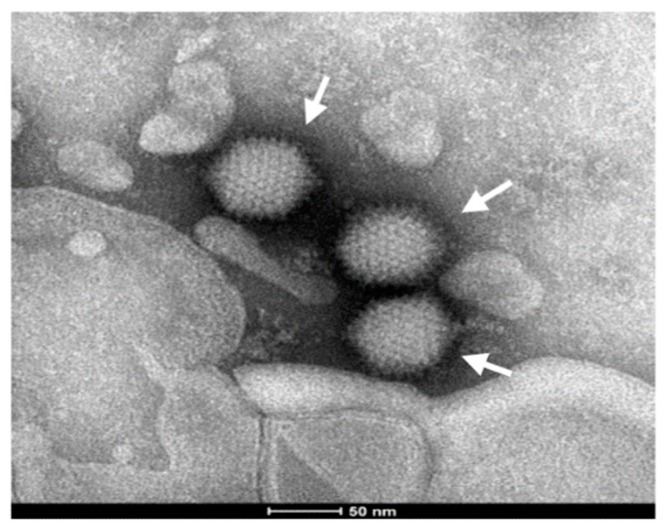
Electron micrograph visualization of pBRAdV14 derived viruses. Scale bar: 50 nm.

The harvested viral culture from pBRAdV14–derived viruses was negative-stained and observed by electron microscopy. Typical adenovirus particles, 70–90 nm in diameter, were clearly found (Figure 7). These further confirmed the infection and replication competence of infectious clone pBRAdV14.

### 3.6. The Analysis of the Sequences of False Positive Infectious Clones

**Figure 8:**
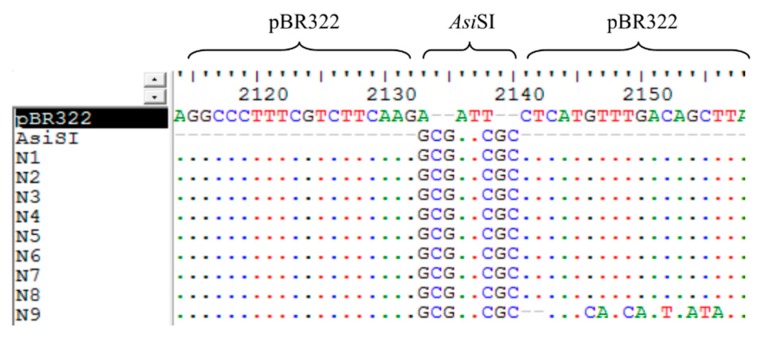
Alignment of the sequences of nine false positive clones with pBR322. The homologous *Asi*SI sequences (GCGATCGC) were recombined into all the false positive clones, while the 40 bp ITR overlapping sequence was deleted. The letters in different color represent the four nucleotides: A in green, T in red, G in black, and C in blue. The dots indicate identical nucleotides.

**Figure 9:**
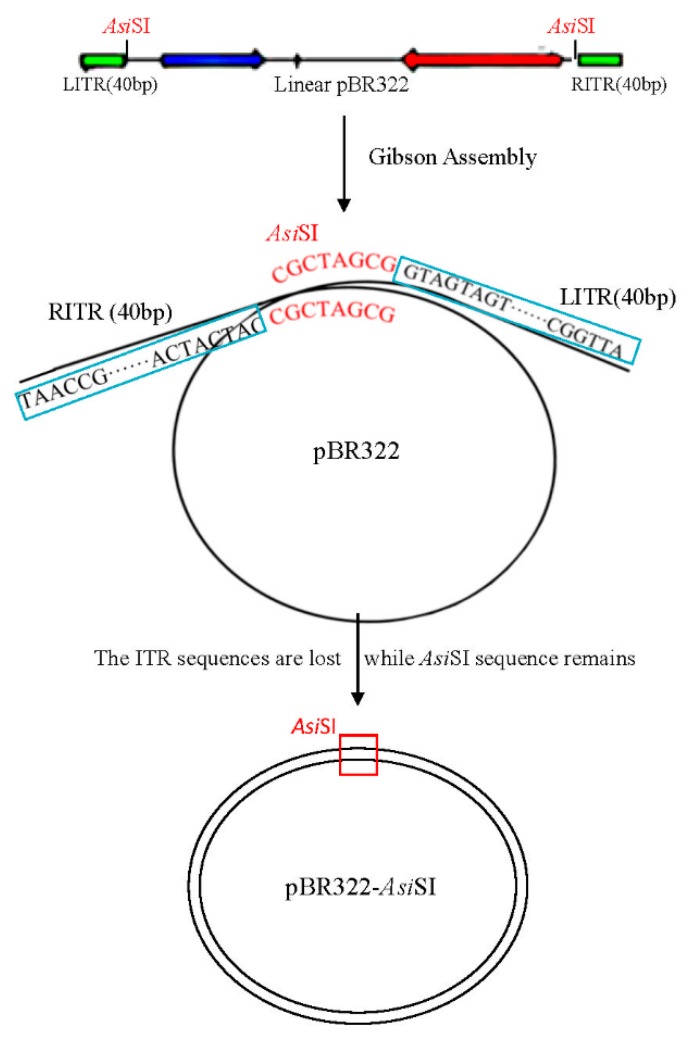
The possible mechanism of the emergence of false positive clones. Linear pBR322 fragment obtained by PCR contained *Asi*SI sites and 40 bp of left ITR (LITR) and right ITR (RITR) sequences. During Gibson Assembly, the homologous region (*Asi*SI sequence) remained while both 40 bp of LITR and RITR were lost. The false positive clones PBR322-AsiSI were produced with *Asi*SI sequence inserted at the linearized site of PBR322.

During the construction of the infectious clones of pBRAdV14, the false positive bacterial clones aroused our attention. The plasmids from the nine negative infectious clones grown in the *Amp*^+^ plates were extracted and the size was similar with the parental pBR322 (~4.3 kb). The plasmid sequences were aligned with pBR322. We found that the sequences of all the nine false positive clones were almost identical with pBR322, except for the insertion of the *Asi*SI sequence (GCGATCGC), while the 40 bp of ITR overlapping sequences were lost, as shown in Figure 8. According to the sequences of these clones, we speculated that during the infectious clone construction, intramolecular homologous recombination within the linear pBR322 fragment containing two *Asi*SI and 40 bp ITR overlapping sequences occurred, which formed a circle plasmid with *Asi*SI sequence insertion (Figure 9). 

## 4. Discussion

HAdV-B14 re-emerged in 2005 and has caused more and more ARD outbreaks with severe and fatal pneumonia in people of all ages, in the United States [15,16,17,18,19,20,21,22,31,32,33], Canada [23], Europe [16,17,18], and Asia [8,11,20,24,25]. The re-emergence of HAdV-B14 has generated extensive concern. Given that HAdV-B14 is one of the two parental viruses contributing to the genome of an emergent ARD pathogen, HAdV-B55 [10,12], it is important to characterize HAdV-B14 for further clarification on the recombination mechanism between HAdV-B14 and HAdV-B11. In addition, no antiviral compounds or vaccines have yet been approved for the treatment and prevention of HAdV-B14 infections. The development of vaccines and drugs against HAdV-B14 is of great importance to human health. As a result, construction of an infectious clone of HAdV-B14 will facilitate further study of gene functions, pathogenic mechanisms, recombination machinery, as well as the development of vaccines and antiviral agents against HAdV-B14. 

During the construction of a replication-competent infectious clone, the major difficulty is that the genome is large, approximately 35 kb. The efficiency and convenience of clone construction is still challenging. Currently, the most common construction strategy for large-genome viruses is to utilize homologous recombination in bacteria, i.e., in *E. coli* BJ5183, GB05-dir, or DH10B pre-transformed with pKD46 [34,35,36,37,38]. However, this method is time-consuming, requiring relatively longer homologous arms (usually more than 500 bp), and requires extensive time for screening and determination of positive bacterial clones. Furthermore, the repeatability is not reliable [38]. For the small-genome viruses, PCR along with ligation or fusion is useful for the infectious clone construction [39,40]. Miciak et al. described a high-copy plasmid-based method that permits individual adenoviral genes to be easily manipulated in isolation and then recombined with other mutations or transgenes [41]. Using this method, they constructed an infectious clone of HAdV-5 and the corresponding mutants without recombination in bacteria. However, attention should be paid to mutations, insertions, or deletions during multiple long-fragment PCR amplifications. In this study, a rapid and efficient in vitro ligation system, Gibson Assembly, which is able to assemble overlapping PCR-amplified DNA molecules in a single isothermal tube [26], was performed successfully to construct an infectious clone of HAdV-B14 (39,140 bp). Firstly, pBR322 was linearized by *Eco*RI digestion and the short overlapping sequences (40 bp of ITRs) were added at the both ends via PCR. Secondly, the resultant pBR322 fragments and the genomic DNA of HAdV-B14 were mixed in a Gibson Assembly reaction tube. After 1-hour incubation at 50 °C, the infectious clone pBRAdV14 was produced. It was transformed into *E. coli* DH5α and grew in the *Amp*^+^ solid medium. The positive bacterial clones were easily screened by PCR. Then the linearized pBRAdV14 was transfected into A549 cells for virus rescue. The total time for the construction and screening can be as short as 3 days, in contrast to 7–14 days by homologous recombination. This construction experiment has been repeated twice. The first time, one positive clone was obtained from four clones. The second time, two positive clones were obtained from eight clones. These demonstrate that Gibson Assembly has favorable repeatability, simple and convenient operability, high efficiency with time saving, and low background, particularly in adenovirus infectious clone construction. Gibson Assembly has also been adopted for infectious clone construction for other pathogens [42,43,44], which shows promising prospect.

Restriction enzyme digestion and the complete sequencing analysis of the infectious clone demonstrated that the infectious clone generated by Gibson Assembly was stable and no mutation occurred during the construction. Based on the one-step growth curves (Figure 7), the DNA replication efficiency of pBRAdV14–derived viruses was almost identical with that of wild-type HAdV-B14 strain GZ01, whereas the infectious viral reproduction was slightly lower. This may be improved by continuing passages of pBRAdV14–derived viruses in A549 cells. Both the sequencing data and replication dynamics indicated that this method has high fidelity in cloning adenoviral genomes when compared with homologous recombination. The bacteria used in homologous recombination usually contain recombinase, which helps the recombination occur efficiently. However, since the size of adenovirus genomes is so big that repeat regions and complex secondary structures are common, the excessive and multiple recombination may occur during the construction [38]. This is adverse for adenovirus clone or vector construction as the fatal mutations may occur because of this excessive recombination. In contrast, there is no recombinase in Gibson Assembly system. Therefore, theoretically, there is no homologous recombination occurring during the construction. 

Given that the plasmid size of the nine false positive clones grown in the *Amp*^+^ LB plates was as small as pBR322, we did the sequencing of these plasmids. The alignment of these sequences with pBR322 showed that *Asi*SI sites (GCGATCGC) remained in the nine false positive clones, whereas the 40 bp ITR overlapping sequences were lost, as shown in Figure 8. The exact mechanism for this is unclear, but according to the sequence alignment, intramolecular homologous recombination might have occurred (Figure 9). The two *Asi*SI sequences within PCR-generated PBR322 fragment can function as homologous arms. During homologous recombination, the *Asi*SI site remained, while the other sequences (40 bp of ITRs) were deleted. This is intriguing as there should not have been recombinase activity in Gibson Assembly reaction, nor is there in host bacteria *E. coli* DH5α. On the other hand, this unexpected finding indicates that Gibson Assembly may be an alternative way to add enzyme recognition sites or other specific sites to a specific location of the plasmid. However, whether recombination occurred during Gibson Assembly or culture in host bacteria *E. coli* DH5α requires further investigation.

The generation of pBRAdV14 lays a foundation for the study of a replication-deficient HAdV-B14 vector, which is significant for vaccine development and gene therapy. Traditional adenovirus vectors are currently based on species C adenovirus HAdV-5 or HAdV-2. However, because of pre-existing immunity in most humans caused by widespread natural infection [45,46], the broad use of traditional adenovirus vectors is limited. HAdV-B14 is an emergent pathogen, and the seroprevalence against it should be low among the population. Therefore, a HAdV-B14–based vector may be an alternative to HAdV-C vectors.

In summary, in order to acquire a tool for the better characterization of the new genomic variant HAdV-B14p1, a full-length infectious genomic clone pBRAdV14 was successfully constructed and characterized using one-step Gibson Assembly. The construction of this replication-competent infectious clone may facilitate the development of vaccine and antiviral drugs against HAdV-B14, and provides a blueprint for rapid construction of infectious viral clones for other DNA viruses.

## Figures and Tables

**Table 1:** Primers used in the study.

Primer	Sequence (5′-3′)	PCR Product (bp)	Purpose
pBR-ITR-F	ATTGGCACCATTCCATCTATAAGGTAT ATTATTGATGATG**gcgatcgc**ctcatgtttgaca gcttatcatcg	4453	Amplification of pBR322 and addition of 5′ ITR homologous arm (40 bp) and *Asi*SI restriction site at both ends
pBR-ITR-R	ATTGGCACCATTCCATCTATAAGGTAT ATTATTGATGATG**gcgatcgc**cttgaagacga aagggcctcg		
pBR-ad14-1-F	CCATCACAAGACAAGCCACA	1334	Screening of positive infectious clones (targeting the left ligation region)
pBR-ad14-1-R	GCCTCAACCTACTACTGGG		
pBR-ad14-2-F	CTTACTG TCATGCCATCCG	1113	Screening of positive infectious clones (targeting the right ligation region)
pBR-ad14-2-R	TGAGTGCCAGCGAGAAGA		
HVR-F	CAGGATGCT TCGGAGTAC	1685	Hexon amplification
HVR-R	TTTCTGAAGTTCCACTCGT		
Fiber-F	CCCTCTTCCCAACTCTGG	1253	Fiber amplification
Fiber-R	GGGGAGGCAAAATAACT		
Penton-F	CTATCAGAATGACCACAG	1153	Penton amplification
Penton-R	TCCCGTG ATCTGTGAGAG		

The underlined sequences: 40 bp of 5′ ITR sequence of HAdV-B14; the sequence in bold: *Asi*SI restriction site.
